# High-Fat Breakfast Increases Bioavailability of Albendazole Compared to Low-Fat Breakfast: Single-Dose Study in Healthy Subjects

**DOI:** 10.3389/fphar.2021.664465

**Published:** 2021-04-15

**Authors:** Dolores Ochoa, Miriam Saiz-Rodríguez, Esperanza González-Rojano, Manuel Román, Sergio Sánchez-Rojas, Aneta Wojnicz, Ana Ruiz-Nuño, Alfredo García-Arieta, Francisco Abad-Santos

**Affiliations:** ^1^ Clinical Pharmacology Department, Hospital Universitario de La Princesa, Instituto Teófilo Hernando, Facultad de Medicina, Universidad Autónoma de Madrid (UAM), Instituto de Investigación Sanitaria La Princesa (IP), Madrid, Spain; ^2^ UICEC Hospital Universitario de La Princesa, Plataforma SCReN (Spanish Clinical Reseach Network), Instituto de Investigación Sanitaria La Princesa (IP), Madrid, Spain; ^3^ Research Unit, Fundación Burgos por La Investigación de La Salud, Hospital Universitario de Burgos, Burgos, Spain; ^4^ Service on Pharmacokinetics and Generics, Division of Pharmacology and Clinical Evaluation, Department of Human Use Medicines, Spanish Agency for Medicines and Health Care Products, Madrid, Spain

**Keywords:** albendazole, breakfast, bioavailability, healthy subjects, pharmacokinetic

## Abstract

**Purpose:** Albendazole is a benzimidazole carbamate drug with anthelmintic and antiprotozoal activity against intestinal and tissue parasites. It has been described that the administration with meals increases albendazole absorption. Our aim was to compare the systemic exposure in healthy volunteers of two albendazole formulations after a single oral dose under fed conditions and to evaluate the effect of breakfast composition on albendazole and albendazole sulfoxide bioavailability.

**Methods:** 12 healthy volunteers were included in a 4-period, 4-sequence, crossover, open, randomized, bioequivalence clinical trial, including two stages to compare two formulations of albendazole. Single oral doses of 400 mg albendazole were administered under fed conditions (a low-fat breakfast in first stage and a high-fat breakfast in the second) separated by 7-day washout periods. Plasma albendazole and albendazole sulfoxide concentrations were measured by HPLC-MS/MS.

**Findings:** Albendazole absorption was clearly influenced by the meal composition. A high-fat breakfast increased albendazole and albendazole sulfoxide area under the concentration–time curve (AUC) and maximum concentration (C_max_) by double, compared to a low-fat breakfast. The bioavailability of the two formulations was very similar, although the sample size was not sufficient to demonstrate bioequivalence because the intraindividual variability of albendazole was approximately 60%.

**Implications:** The higher albendazole and albendazole sulfoxide levels when administered with a high-fat meal could be of importance in clinical practice. Since albendazole labeling recommends its administration with meals, it is necessary to insist on taking it with a fatty meal so that the effectiveness of albendazole is not compromised.

## Introduction

Albendazole is a benzimidazole carbamate drug with anthelmintic and antiprotozoal activity against intestinal and tissue parasites. It is especially indicated for the treatment of systemic helminthic infections such as echinococcosis (hydatid disease) with hepatic, lung or peritoneal cysts, and neurocysticercosis. Albendazole acts by binding to parasite tubulin, thus inhibiting its polymerization (Venkatesan, 1998).

Albendazole absolute oral bioavailability is less than 5% in humans. After oral administration, it rapidly undergoes first-pass metabolism in the liver. Its primary metabolite, albendazole sulfoxide, is considered the active moiety against tissue infections. Its plasma half-life (T_1/2_) is around 9 h. Moreover, it is 70% protein bound (Venkatesan, 1998). Albendazole and its metabolite seem to be eliminated primarily in bile, with only a small portion excreted in urine. However, a study in human liver microsomes and recombinant cytochrome P450 enzymes (CYP) showed for the first time that albendazole hydroxylation is primarily catalyzed by CYP2J2 (Wu et al., 2013).

Pharmacokinetic parameters of albendazole are dose-dependent, explained by a slow and incomplete dissolution in the gastrointestinal tract (Mirfazaelian et al., 2002a). After a single oral administration of 400 mg of albendazole with fatty breakfast, the active metabolite reaches plasma concentrations of 425–1,592 ng/ml. In addition, when administered along with a high-fat meal, albendazole increased its absorption approximately 5 times compared to the administration under fasting conditions (Lange et al., 1988). Indeed, Schmidt and Dalhoff described a clinically relevant interaction between a fatty meal and albendazole administration, especially for the treatment of systemic infections (Schmidt and Dalhoff, 2002). Moreover, albendazole properties were enhanced by its administration with a fatty breakfast in patients with onchocerciasis (Awadzi et al., 1994). It is of importance to highlight that these studies compare high-fat meals with fasting conditions. None of them compared high-fat food with low-fat meals, but in the usual practice, patients could take albendazole with a low-fat breakfast. Indeed, albendazole is widely used to treat parasitic infections that predominate in tropical and subtropical regions where local diet is highly variable, and it may also be used in inpatient and outpatient settings where concomitant diet may differ.

This study aimed to compare the systemic exposure in healthy volunteers of two albendazole formulations after a single oral dose under fed conditions and to evaluate the effect of the breakfast composition on its bioavailability.

## Materials and Methods

### Methods

#### Study Population

Our study population comprised 12 healthy volunteers included in a comparative bioavailability trial performed at the Clinical Trials Unit of Hospital Universitario de La Princesa (Madrid, Spain).

The protocol complied with current Spanish legislation on clinical research in humans and was approved by the Research Ethics Committee, duly authorized by the Spanish Agency for Medicines and Health Care Products and under the guidelines of Good Clinical Practice (EUDRA-CT: 2010–021006–38, registered on 13/10/2011). All subjects gave their written informed consent and were free to withdraw from the study at any time.

The inclusion criteria were as follows: nonsmoking male and female volunteers, age 18–55 years, body mass index (BMI) within the 18.5–30.0 range, free from any organic or psychiatric conditions, with normal vital signs, electrocardiogram (ECG), medical records, and physical examination. Healthy volunteers had no gastrointestinal tract or liver diseases and had normal liver functions. It was not allowed to take other drugs, grapefruit juice, or ginseng or herbal products during the study.

#### Study Design and Procedures

The study included two consecutive stages with a crossover, open and randomized design, performed in the same subjects. Treatment assignment was made by randomization in balanced blocks of 4 individuals, with a table of random numbers, to maintain a classic 4-period, 4-sequence crossover design. Single oral doses of 400 mg albendazole were administered under fed conditions including a low-fat Spanish breakfast in the first stage (periods 1 and 2) and a high-fat, high-calorie breakfast in the second stage (periods 3 and 4), separated by washout periods of 7 days between administrations. We compared two albendazole formulations marketed by the same company (Eskazole® new or test formulation and old or reference formulation) that differ in the disintegrant and the amount of some excipients, including the amount of a critical excipient (surfactant), and the manufacturing process. The person responsible for measuring the concentrations of albendazole and albendazole sulfoxide did not know which formulation the subject had received or under what conditions.

After physical examination, vital signs were recorded and electrocardiogram (ECG) was performed. Then, a low-fat breakfast or a high-fat breakfast was given, after fasting for at least 10 h (see Table 1 for composition of each breakfast). Breakfast was consumed in a maximum of 20 and 10 min later a single dose of the corresponding albendazole formulation was administered.

**TABLE 1:** Content of the low-fat and high-fat breakfasts.

Low-fat breakfast					High-fat breakfast				
Food	Proteins	Carbohydrates	Fat	Kcal	Food	Proteins	Carbohydrates	Fat	Kcal
White bread (100 g)	8	55.9	0.6	236	White bread (60 g)	4.7	34.8	0.6	163.2
Whole milk (200 ml)	6.6	10	7.2	132	Whole milk (200 ml)	6.6	10	7.2	132
Jam (30 g)	0.06	0.68	0	4	Oil* (30 g)			30	270
Water (200 ml)					One egg (80 g)	10		8.9	120
					French fries (100 g)	2.5	18	0.2	84
					Sausage (50 g)	11	1	10.5	142.5
					Water (200 ml)				
Total kcal	58.64	246.35	70.2	375.19	Total kcal	139.2	255.2	516.6	911.7
Relative caloric content (%)	15.63%	65.66%	18.71%	100%	Relative caloric content (%)	15.3%	28%	56.7%	100%

* Oil was used for frying the egg, sausage, and French fries.

Albendazole was administered by oral route with 240 ml of water. For the pharmacokinetic analysis, 20 blood samples were obtained at baseline (before the drug was administered) 0.5, 1, 1.5, 2, 2.5, 3, 3.5, 4, 4.5, 5, 5.5, 6, 7, 8, 11, 15, 19, 24, and 32 h after tablets administration. Samples were centrifuged at 4°C for 10 min at 3,500 rpm (1900 g). Quantification of plasma albendazole and albendazole sulfoxide concentrations was performed by reversed phase high performance liquid chromatography and detected by tandem mass spectrometry (HPLC-MS/MS), as previously validated by our group (Wojnicz et al., 2013), according to European Medicines Agency regulatory requirements (European Medicines Agency). Briefly, each plasma sample was extracted by solid phase extraction (SPE) using phenacetin as internal standard (IS) (Wojnicz et al., 2013). The extracted sample was eluted with a Zorbax XDB-CN column using an isocratic method. The mobile phase consisting of water with 1% acetic acid (40%, A) and MeOH (60%, B) was used at a flow rate of 1 ml/min (Wojnicz et al., 2013). Albendazole and albendazole sulfoxide were detected and identified by mass spectrometry with electrospray ionization (ESI) in the positive ion and multiple-reaction monitoring (MRM) mode (Wojnicz et al., 2013). The method was linear in the range of 5–1,000 ng/ml for albendazole and 10–1,500 ng/ml (full validation) or 10–5,000 ng/ml (partial validation) for albendazole sulfoxide, with 5 and 10 ng/ml lower limit of quantification (LLOQ) for albendazole and albendazole sulfoxide, respectively (Wojnicz et al., 2013).

#### Pharmacokinetic Analysis

Pharmacokinetic parameters were calculated by non-compartmental methods using WinNonlin Professional, version 2.0 (Pharsight Corporation, Palo Alto, California). Maximum concentration (C_max_) and time to reach the C_max_ (T_max_) were directly obtained from raw data (Saiz-Rodríguez et al., 2020). Area under the concentration–time curve (AUC) was calculated from the administration to the last measured concentration (AUC_t_) by linear trapezoidal integration (Saiz-Rodríguez et al., 2020). The total AUC from administration to infinity (AUC_∞_) was calculated as the sum of AUC_t_ and the residual area (C_t_ divided by k_e_, with C_t_ as the last measured concentration and k_e_ as the apparent terminal elimination rate, which was estimated by log-linear regression from the terminal portion of the log-transformed concentration–time plots) (Saiz-Rodríguez et al., 2020). Half-life (T_1/2_) was calculated by dividing 0.693 by k_e_ (Saiz-Rodríguez et al., 2020).

#### Sample Size Calculation

There was no available data in the literature for calculating intraindividual coefficients of variation. Therefore, we considered a 20–22% intraindividual variability for albendazole pharmacokinetic parameters, so the sample size necessary to obtain a 80% statistical power with a replicate design and α = 0.05 within the limits of acceptance (80–125%) would be 8–12 volunteers, with a <5% difference between the two formulation means (Julious, 2004). In addition, with 12 volunteers, and a 80% statistical power, differences of 30% in bioavailability between the low-fat and high-fat breakfast would be achieved.

#### Statistical Analysis

The bioavailability comparison of both albendazole formulations was done with the statistical package integrated in the pharmacokinetic program WinNonlin Professional Edition, version 2.0 (Scientific Consulting, Inc., Cary, United States). The primary kinetic parameters AUC_t_ and C_max_ were transformed logarithmically, and the 90% confidence intervals (CIs) for the ratio test/reference were calculated. The analysis of variance (ANOVA) performed takes into account 4 factors: sequence, subject (sequence), period, and formulation. In the first approach, bioavailability analysis between formulations was performed separately for each type of breakfast (low-fat breakfast or high-fat breakfast).

The effect of sex and other factors was assessed considering all the available data with an ANOVA model including sex, sequence, sex*sequence, subject (sex*sequence), type of breakfast, period, formulation, type of breakfast*formulation, and sex*formulation, similar to previous studies (González‐Rojano et al., 2019). The effect of sex was calculated using subject (sex*sequence) as error term. As there was no interaction between factors nor sex effect, we evaluated the bioequivalence considering all the available data, using a simpler ANOVA model that includes the following factors: sequence, subject (sequence), type of breakfast, period, and formulation. In this way, we could also calculate the ratio and 90% CI of high-fat breakfast compared to low-fat breakfast.

According to U.S. Food and Drug Administration (FDA) and European Medicines Agency (EMA), the formulations were considered bioequivalent if the 90% CI of the ratio test/reference for AUC and C_max_ fell between 80 and 125% (FDA (Food and Drugs Administration), 2001; FDA (Food and Drugs Administration), 2003; EMA (European Medicines Agency), 2011; European Medicines Agency, 2010). Intra-subject variability, expressed as percent coefficient of variation (CV), was estimated by the mean square error from ANOVA according to the EMA guidelines (European Medicines Agency, 2010).

## Results

### Study Subjects

From 18 informed volunteers, only 16 signed the written informed consent and 12 (9 men and 3 women) were included in the study. All of them completed the study and were included in the pharmacokinetic analysis. Mean demographic data of the included subjects are shown in Table 2. All subjects were Caucasians.

**TABLE 2:** Main demographic characteristics of the included healthy volunteers.

	Age (years)	Weight (kg)	Height (m)	BMI (kg/m^2^)
Men (n = 9)	24.6 (4.8)	78.0 (4.7)	1.82 (0.05)	23.7 (1.3)
Women (n = 3)	23.2 (1.7)	62.0 (8.1)*	1.67 (0.01)*	22.1 (3.0)

Values are shown as mean (SD). *p < 0.05, BMI, body mass index.

### Albendazole and Albendazole Sulfoxide Pharmacokinetics

**FIGURE 1:**
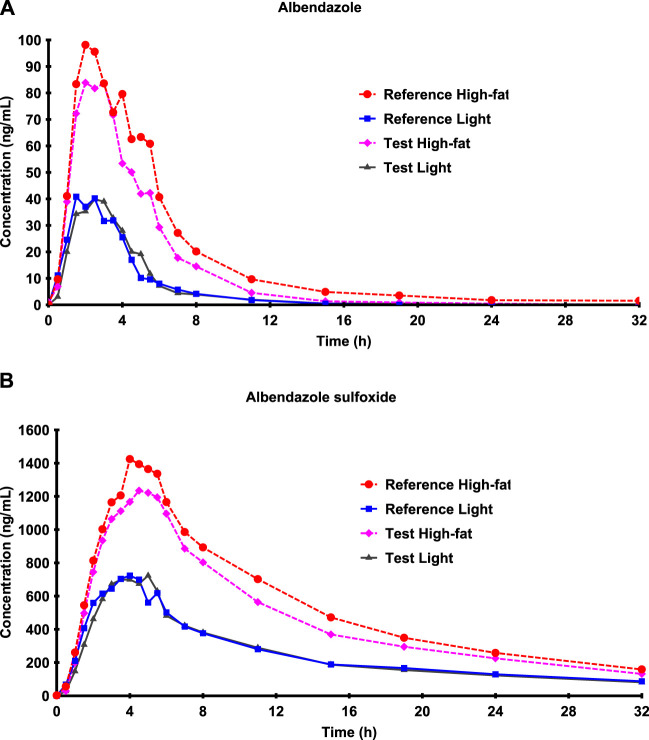
Mean albendazole **(A)** and albendazole sulfoxide **(B)** concentration–time curves after low-fat and high-fat breakfast intake on a linear scale.

The mean concentration–time curves of albendazole were very similar for the two formulations (Figure 1A) after low-fat and high-fat breakfast intake. However, concentrations were more than 2-fold higher after high-fat than after low-fat breakfast. The mean concentration–time curves of albendazole sulfoxide were also similar for the two formulations, and differences between low-fat and high-fat breakfast were noticeable as well (Figure 1B).

The influence of food on albendazole absorption can be also observed in the pharmacokinetic parameters of both formulations (Table 3). All pharmacokinetic parameters for albendazole and albendazole sulfoxide were similar between formulations, but they differ according to the type of breakfast administered. On average, terminal albendazole T_1/2_ was calculated as about 2–3 h for low-fat and high-fat food for both products.

**TABLE 3:** Albendazole and albendazole sulfoxide mean pharmacokinetic parameters for both formulations according to the type of breakfast.

		AUC_∞_ (ng·h/mL)	AUC_t_ (ng·h/mL)	AUC % extrapolated	C_max_ (ng/ml)	T_max_ (h)	T_1/2_ (h)
Albendazole							
Low-fat breakfast	Reference	202.4 (174.3)	156.3 (164.0)	16.3 (11.6)	57.5 (52.3)	1.75 (1.00–4.50)	2.0 (1.1)
	Test	265.3 (222.8)	163.3 (200.8)	16.2 (16.5)	53.4 (51.4)	2.50 (1.50–5.00)	3.0 (2.0)
High-fat breakfast	Reference	642.9 (762.8)	550.3 (728.5)	15.1 (17.0)	128.9 (160.4)	2.50 (2.00–5.50)	2.8 (1.5)
	Test	429.8 (389.5)	397.1 (382.9)	11.8 (9.5)	123.9 (103.9)	2.25 (1.50–5.50)	2.7 (1.6)
**Albendazole sulfoxide**							
Low-fat breakfast	Reference	9,867.1 (6,180.9)	8,125.4 (4,761.4)	15.3 (6.8)	830.0 (439.9)	3.50 (2.00–5.50)	12.6 (4.2)
	Test	9,489.4 (6,581.2)	7,934.1 (5,108.7)	14.7 (6.4)	860.6 (436.2)	3.50 (2.00–5.50)	12.3 (3.2)
High-fat breakfast	Reference	19,919.9 (16,139.5)	17,157.0 (13,676.4)	11.9 (7.4)	1,580.2 (1,080.2)	4.00 (3.50–5.50)	11.0 (4.8)
	Test	17,020.9 (10,302.3)	14,781.2 (8,164.4)	11.5 (6.0)	1,371.7 (578.7)	3.50 (2.00–5.50)	10.5 (3.2)

Values are shown as mean (standard deviation) but median (range) for Tmax. Abbreviations: AUC_∞_, area under the concentration–time curve from administration time to infinity; AUC_t_, area under the concentration–time curve from administration time to the last measured concentration at 32 h; C_max_, maximum concentration; T_max_, time to reach the maximum concentration; T_1/2_, half-life. %AUC extrapolated = AUC_∞_ - AUC_t_/AUC_∞_.

ANOVA did not show significant differences between the two formulations for any albendazole pharmacokinetic parameter (*p* = 0.5723 for AUC_t_ and *p* = 0.6903 for C_max_), but AUC and C_max_ were significantly higher when administered with high-fat breakfast (*p* < 0.0001). There were no differences in T_max_ (*p* = 0.1766) or half-life (*p* = 0.1567) according to the type of breakfast. Ratio (90%CI) of high-fat breakfast compared to low-fat breakfast was 377.80% (266.31–535.95) for AUC_t_ and 234.08 (188.15–291.23) for C_max_. There were not significant differences between men and women (*p* = 0.5976 for AUC_t_, *p* = 0.7,338 for C_max_, *p* = 0.3057 for T_max_, and *p* = 0.4822 for half-life).

The extrapolated percentage of AUC for albendazole under low-fat breakfast was on average 16.3% and ranged between 4.4 and 55.9%, being higher than 20% (limit set by regulatory authorities) in four subjects after reference and in one subject after test formulation intake. Under high-fat breakfast, it was on average 13.5% and ranged between 1.6 and 64.0%, being higher than 20% in two subjects after reference and two subjects after test formulation intake. With respect to albendazole sulfoxide, the extrapolated AUC was lower than 20% in all subjects but two under low-fat breakfast and one after high-fat breakfast (Table 3).

All albendazole sulfoxide pharmacokinetic parameters were similar to those previously described in the literature (Lange et al., 1988; Rigter et al., 2004; Edi et al., 2019) and showed similar results for both formulations (Table 3). ANOVA did not show significant differences between the two formulations for any parameter, but there were significant differences between the type of breakfast for AUC and C_max_ (*p* < 0.0001). Ratio (90%CI) of high-fat breakfast compared to low-fat breakfast was 199.35% (170.89–232.55) for AUC_t_ and 176.43 (149.54–208.16) for C_max_. T_max_ was shorter after low-fat breakfast (mean 3.6 vs 4.2 h, *p* = 0.0034), and half-life was shorter after high-fat breakfast (12.4 vs 10.8 h, *p* = 0.0363). There were no differences between men and women for any parameter (*p* = 0.7610 for AUC_t_, *p* = 0.7669 for C_max_, *p* = 0.2918 for T_max_, and *p* = 0.6526 for half-life).

The intra-subject variability in the main pharmacokinetic parameters of albendazole (AUC and C_max_) was very high after both low-fat and high-fat breakfast consumption (Table 4). For albendazole sulfoxide, the intra-subject variability was lower than for albendazole after both low-fat and high-fat breakfast (Table 4).

**TABLE 4:** Main results concerning bioequivalence assessment for albendazole and albendazole sulfoxide considering the classical ANOVA model: sequence, subject (sequence), period, and formulation. Ratio (test/reference) and 90% confidence interval.

Low-fat breakfast (n = 12)				
Parameter	Albendazole	CV (%)	Albendazole sulfoxide	CV (%)
C_max_	91.40 (67.41–123.92)	42.9	103.39 (84.36–126.72)	28.0
AUC_t_	84.14 (56.08–126.26)	59.2	95.09 (81.47–110–99)	21.1
**High-fat breakfast (n = 12)**				
**Parameter**	**Albendazole**	**CV (%)**	**Albendazole sulfoxide**	**CV (%)**
C_max_	121.38 (90.56–162.68)	41.2	99.12 (76.08–129.13)	36.9
AUC_t_	101.49 (66.85–154.06)	61.2	98.75 (77.50–125.82)	33.6

Abbreviation: CV, intra-subject coefficient of variation.

Regarding the comparative bioavailability analysis with both low-fat and high-fat breakfasts, bioequivalence was not demonstrated for albendazole or albendazole sulfoxide because the 90% confidence interval for the corresponding mean ratios (test over reference) of AUC_t_ and C_max_ was not contained within the predefined bioequivalence acceptance range of 80.00–125.00 (Table 4). However, if both studies are analyzed together, bioequivalence is reached for albendazole sulfoxide (Table 5) although intra-subject variability is not reduced.

**TABLE 5:** Analysis of bioequivalence considering an ANOVA model that includes sequence, subject (sequence), type of breakfast, period, and formulation. Ratio (test/reference) and 90% confidence interval.

Albendazole (n = 12)			
Parameter	Ratio	Confidence interval (90%)	CV
C_max_	105.33	85.06–130.43	45.9
AUC_t_	92.41	64.73–131.92	83.6
**Albendazole sulfoxide (n = 12)**			
**Parameter**	**Ratio**	**Confidence interval (90%)**	**CV (%)**
C_max_	101.23	85.57–119.76	35.4
AUC_t_	96.91	82.91–113.27	32.7

Abbreviation: CV, intra-subject coefficient of variation.

## Discussion

It is worth noting that the bioavailability of albendazole and its metabolite was much higher when administered after a high-fat breakfast than after a low-fat breakfast, with an increase in AUC of almost 4 times for albendazole and 2 times for albendazole sulfoxide. Our results are consistent with those previously described by several groups about the increase of albendazole absorption when administered along with a fatty meal (Lange et al., 1988; Awadzi et al., 1994; Schmidt and Dalhoff, 2002; Mares et al., 2005). However, these studies compared the bioavailability of albendazole administered with high-fat meals with the administration under fasting conditions. According to the product labeling (FDA (Food and Drugs Administration)), plasma concentrations of albendazole sulfoxide are up to 5-fold higher on average when albendazole is administered with a fatty meal (fat content of approximately 40 g) than administration in fasting conditions. In one study of six healthy male volunteers (Awadzi et al., 1994), administration of a single 10 mg/kg oral dose of albendazole in combination with a high-fat meal (57 g fat, 1,399 kcal) increased the mean albendazole sulfoxide C_max_ and systemic exposure (AUC) by 6.5- and 9.4-fold, respectively, and delayed T_max_ from 2.5 to 5.3 h compared to administration in the fasted state with water. Other study performed with 16 healthy volunteers showed that mean C_max_ and AUC value were enhanced 7 times after the administration of a Mexican diet (fat 57.1%, protein 16%, carbohydrates 26%, and 963.5 kcal) (Mares et al., 2005), a significant delay in T_max_ was observed (from 3.16 to 5.13 h for fasting and fed conditions, respectively), but the elimination half-life was not affected.

According to our knowledge, our study is the first to compare the impact of a high-fat vs. a low-fat breakfast. In our study, the total fat content in low-fat breakfast was 7.8 g and in high-fat breakfast was 57.4 g. Hence, differences related to bioavailability are expected. Of note, albendazole labeling recommends its administration with meals, but it does not specify the type of meal, so the fat content of patients’ diet could be relevant. Patients usually take albendazole with a low-fat breakfast. Therefore, clinicians should focus on the type of meal the patients are taken with this drug, since differences in bioavailability could have an impact on albendazole efficacy.

Another study in 8 healthy volunteers who were given 400 mg albendazole with a standard meal (fat content 40 g) found an albendazole sulfoxide C_max_ of around 1,200 ng/ml, which is in between the two types of meal that we evaluated (Ceballos et al., 2018). However, AUC was higher in this study because they found a higher half-life (14.5 8) than ours (around 11 h after high-fat and around 12 h after low-fat breakfast).

In another study (Schulz et al., 2019), ten hookworm-infected adolescents (from 15 to 20 years of age) were orally treated with 400 mg albendazole and 25 mg/kg oxantel pamoate. They received oily fish on bread as a standardized breakfast before treatment, but they got very low concentrations of albendazole in plasma (24.5 ng/ml) and albendazole sulfoxide (288 ng/ml), around 20% of the concentrations that we got after high-fat breakfast, which can limit the efficacy of treatment. This might be due to the low-fat content of the administered breakfast, which cannot be confirmed because the authors did not provide details on its composition.

Food enhances albendazole oral bioavailability, which, after absorption, is rapidly converted by enterocytes and hepatocytes into the active metabolite, albendazole sulfoxide. This fact might be explained by the stimulation of gastric acid secretion as the absorption of albendazole is thought to be pH-dependent (Durgs), due to its higher solubility at acidic pH. In fact, cimetidine decreased the C_max_ of the active metabolite by 52% and the AUC by 28% due to its effect on gastric pH, and it prolonged the elimination half-life of the active metabolite as CYP inhibitor (Schipper et al., 2000). However, as food also increases pH, in our opinion, the increased absorption is mainly caused by the stimulation of bile secretion or food-induced increase of drug solubility (Schmidt and Dalhoff, 2002). The increased dose absorbed could be related to a higher dissolution of albendazole in the gastrointestinal tract, probably due to the surfactant effect of the bile salts which are secreted after the meal (Mares et al., 2005). Previous *in vitro* studies have shown that solubility, but not absorption, was the rate-limiting step in the bioavailability of albendazole (Jung et al., 1998).

Interestingly, in our study, the increase of albendazole plasma levels is proportionally higher than those of the active metabolite. This could be explained by the fat content of meals, which might decrease first-pass metabolism (Melander and McLean, 1983). Consequently, a higher amount of parent drug is absorbed intact, and less active metabolite is formed in the first-pass step. It was suggested that grapefruit juice increases the oral bioavailability of albendazole sulfoxide by inhibiting CYP3A4: in six healthy male volunteers, administration of a single 10 mg/kg oral dose of albendazole in combination with 250 ml of double-strength grapefruit juice increased the mean albendazole sulfoxide C_max_ and AUC by 3.2- and 3.1-fold, respectively, compared to administration with water (Nagy et al., 2002). However, these authors only measured the plasma levels of the active metabolite, and, if intestinal formation of albendazole metabolite was inhibited by grapefruit, the reduction of albendazole sulfoxide plasma levels would be expected. Grapefruit juice also activates P-glycoprotein–mediated efflux of drugs (Soldner et al., 1999), which would increase the exposure time of albendazole to the intestinal CYPs and increase its metabolism. Importantly, these authors could not explain the unexpected finding of a 46% shortening of the metabolite half-life, which may be related to the assumption of no systemic exposure to albendazole, since it was not detected by their bioanalytical method, but it can be detected, as shown in our study.

The increase in plasma levels of albendazole sulfoxide did not decrease the interindividual variability; therefore, it can be assumed that the main cause of variability is due to differences in gastric pH or intestinal metabolism (Schipper et al., 2000; Nagy et al., 2002; Mares et al., 2005).

Another factor to take into account is the interaction between albendazole and praziquantel, which was demonstrated in healthy volunteers (Lima et al., 2011). The combination of these two drugs is of clinical importance, since it increased albendazole sulfoxide plasma concentrations, which might improve the therapeutic efficacy (Lima et al., 2011). Although the mechanism of this interaction is unclear, it was suggested that it may be mediated by P-glycoprotein. However, there are no data in the literature demonstrating that albendazole and/or its metabolites are substrates of intestinal P-glycoprotein in humans. In any case, when administered with high-fat breakfast, this type of plasma concentrations elevation might develop an increased risk of adverse drug reactions.

Another study that compared the pharmacokinetics of albendazole in healthy male and female volunteers (Mirfazaelian et al., 2002b) found no significant differences in albendazole sulfoxide T_1/2_ and T_max_, although clearance and volume of distribution were lower in women (Mirfazaelian et al., 2002b). On the contrary, C_max_ and AUC were higher in women (Mirfazaelian et al., 2002b). Mirfazaelian *et al.* stated that these differences were a result of a more extensive albendazole first-pass metabolism in women (Mirfazaelian et al., 2002b). In our study, we found no significant differences in any of the pharmacokinetic parameters of albendazole and albendazole sulfoxide between sexes, but it might be explained by the low number of subjects.

Regarding albendazole and albendazole sulfoxide pharmacokinetics, we did not find any statistically significant difference between both formulations for AUC or C_max_. However, our small sample size was too small to detect differences and to demonstrate bioequivalence. We failed in our purpose probably due to the lack of information regarding the intraindividual variability of albendazole when the study was conducted. Our assumption of intra-subject variability of the pharmacokinetic parameters of 20–22% was far from reality. The observed intra-subject variability for albendazole was around 60%, for both types of breakfast, so the sample size needed should be larger than 60 volunteers in a replicate design (Julious, 2004).

However, both formulations could be considered therapeutic equivalent since albendazole sulfoxide is the one exerting the main pharmacological effect, and its AUC and C_max_ confidence intervals were between 80 and 125% if we considered all the information obtained in this study. According to the EMA guideline (European Medicines Agency, 2010), evaluation of bioequivalence should be based upon measured concentrations of the parent compound. However, some prodrugs may have low plasma concentrations and might be quickly eliminated, resulting in difficulties in demonstrating bioequivalence for the parent compound, as might happened with albendazole. In this situation, it is acceptable to demonstrate bioequivalence for the main active metabolite without measurement of the parent compound. Importantly, the old and the new formulations of the innovator product were compared based on *in vitro* dissolution profiles only, when the marketed formulation was changed worldwide. This study is the only *in vivo* comparison available to support that the formulation change does not alter significantly the bioavailability and, consequently, the efficacy and safety of the product.

According to the EMA guideline on the investigation of bioequivalence (European Medicines Agency, 2010), in studies determining bioequivalence after a single dose, the parameters to be analyzed are AUCt and C_max_. Thus, the 90% confidence interval for AUCinf is not usually calculated, mainly because it is a parameter that can be easily modified according to the procedure used to calculate the terminal elimination rate. Moreover, according to EMA guideline, AUCt should cover at least 80% of AUC_∞_ (European Medicines Agency, 2010). If the percentage is less than 80% in more than 20% of the observations, the validity of the study may be compromised. It occurs for albendazole after receiving a low-fat breakfast due to the low concentrations reached; so, this condition is not appropriate for evaluation of bioequivalence. Indeed, the EMA guideline recommends the administration of a high-fat breakfast for evaluation of bioequivalence under fed conditions, where the number of subjects with extrapolated AUC higher than 20% is lower than 20%.

## Conclusion

Albendazole absorption is clearly influenced by the type of meal. A high-fat breakfast increases albendazole sulfoxide AUC_t_ and C_max_ twofold. This could be extremely important in clinical practice. Since albendazole labeling recommends its administration with meals, it is necessary to control the patient's diet and its fat content so that the effectiveness of albendazole is not affected. Regarding the bioequivalence of the two formulations, their bioavailabilities were very similar, although the sample size was not sufficient to demonstrate bioequivalence in the two types of meal separately because the intraindividual variability of albendazole was approximately 60%. Nevertheless, the whole dataset was able to show bioequivalence for the active metabolite, which ensures the therapeutic equivalence.

The data that support the findings of this study are available on request from the corresponding author. The data are not publicly available due to privacy or ethical restrictions.

## Data Availability

The raw data supporting the conclusions of this article will be made available by the authors, without undue reservation.
